# Designing, refining and reflecting on 3 years of daily evidence surveillance for Australia's living national COVID‐19 guidelines

**DOI:** 10.1002/cesm.12045

**Published:** 2024-02-26

**Authors:** Steve McDonald, Tari Turner

**Affiliations:** ^1^ Cochrane Australia, School of Public Health and Preventive Medicine Monash University Australia

**Keywords:** Australia, COVID‐19, evidence surveillance, guideline development, living guidelines, search strategies

## Abstract

Australia's national living guidelines for COVID‐19 were launched in April 2020 and include over 200 recommendations to guide the clinical care of patients with COVID‐19. Until the guidelines were retired in August 2023, new evidence was continually monitored through daily surveillance. This paper describes the initial design for evidence surveillance (at a time when efforts to collate information on the novel coronavirus were in their infancy and often duplicative) and how it was refined throughout the pandemic. Among the wide range of sources monitored, the collections of COVID‐19 research from leading medical journals, medRxiv preprints and PubMed auto alerts proved the most enduring in identifying time‐critical and impactful evidence. The paper also explores how evidence was tracked and surveillance integrated into the overall evidence workflow by using messaging apps and communication platforms. Finally, we consider the implications for living guidelines and reflect on factors that contributed to the feasibility of daily surveillance: the clearly defined scope of the guidelines; focusing efforts on maximum impact; minimizing duplication by partnering with others; setting up simple but effective processes for managing evidence; and the value of continuous involvement of personnel from the outset. Australia's living COVID‐19 guidelines were underpinned by a novel approach to evidence surveillance that was feasible and essential in maintaining COVID‐19 living guidelines for over 3 years.

## 1 INTRODUCTION

Early in 2020 the Australian National Clinical Evidence Taskforce (NCET) was established with the ambitious purpose of providing clinicians with living, evidence‐based recommendations for the clinical care of patients with COVID‐19. At the end of March 2020, with the first tranche of 19 member organizations on board and guideline panels appointed, the first draft recommendations were agreed.

Version one of the GRADE‐based guidelines was published on April 3, 2020 via the MAGICapp platform and included nine consensus‐based recommendations. The 74th version was published in May 2023 and included over 200 recommendations [1]. In the intervening 3 years, 36 member organizations had joined the Taskforce and the guidelines had been viewed over 1.6 million times. As of August 2023 the guidelines were no longer being updated.

The speed at which the pandemic took hold and the near absence of research evidence to inform decisions in the initial period challenged orthodox principles of searching such as transparency and rigor. There was no established roadmap for accessing information about a disease that had barely been identified and defined, and whose consequences for individuals and society were unknown. Like others who were trying to develop guidance, the approach adopted was necessarily pragmatic.

From the outset, as it became clear that weekly updates to the guidelines would be required, daily evidence surveillance—the process of continually identifying relevant studies—was key to keeping the guidelines up to date. Several papers have described the development, content and impact of the Taskforce COVID‐19 guidelines [2, 3, 4, 5, 6, 7]. This paper focuses on the search and surveillance methods for the living guidelines—how daily evidence surveillance was established and evolved during the pandemic, and how it was integrated into the overall guideline workflow—and includes reflections on feasibility and implications.

## 2 EVERYTHING EVERYWHERE ALL AT ONCE

The early phase of the pandemic saw an unparalleled wave of research relating to the coronavirus emergency and a scramble to collate, organize and synthesize information. For example, publications in LitCovid (https://www.ncbi.nlm.nih.gov/research/coronavirus/), an open database of COVID‐19 literature maintained by the US National Library of Medicine, increased six‐fold between March and May 2020, from 50 to over 300 publications a day. The need to access up‐to‐date research rapidly and efficiently, together with the proliferation of new information sources dedicated to the novel coronavirus in the space of a few weeks, presented new challenges to information specialists responsible for evidence searches for guidelines and other syntheses.

At the same time as organizations like the US National Library of Medicine [8], World Health Organization, and US Centers for Disease Control & Prevention started to compile databases of all COVID‐19 research, groups like Cochrane [9] and Epistemonikos [10] developed registers of COVID‐19 studies, and others such as US Veterans' Affairs Evidence Synthesis Program established an inventory of reviews [11]. Alongside these initiatives were groups providing living syntheses of evidence (e.g., COVID‐NMA initiative [12]) and living maps of COVID‐19 research (e.g., EPPI‐Centre [13]).

Although some excellent guides were compiled as the pandemic unfolded (e.g., COVID‐END guide to key evidence sources [14]), these were lacking in the early days. When daily surveillance was set up, there was little time to appraise and compare resources—the choice of one over another was a mix of serendipity, reputation, ease of use and personal preference.

Our initial focus was on developing guidance around clinical signs and symptoms of SARS‐CoV‐2 and recommendations for the care of people with confirmed or suspected COVID‐19. In those early days, faced with several sources of substantially overlapping coronavirus research, a key consideration in setting up daily surveillance was the ability to easily identify newly added research. For this reason, the US Centers for Disease Control & Prevention (CDC) Database of COVID‐19 Research Articles [15] was initially selected as the main source. A RIS file, a tagged format for expressing bibliographic citations, of new records was made available daily on the CDC website and records relevant to the initial guideline topics (primarily around drug treatments and respiratory support interventions) were screened in EndNote. Other factors in choosing the CDC database were its comprehensive coverage of traditional and gray literature sources, and transparency around its compilation.

## 3 IT WAS SEARCHING BUT NOT AS WE KNEW IT

In contrast to evidence surveillance for standard guidelines, which typically relies on bibliographic database searching, the speed and way research was disseminated during the pandemic impacted the activities and scope of the search surveillance. The contrast was most evident in three areas—preprints, media releases and retractions.

The emergence of preprints was a new phenomenon for clinical guidelines [16], and policies were hastily drafted that set out the conditions under which preprint data would be considered in the development of guideline recommendations. At the outset of the pandemic, preprints were not routinely included in bibliographic databases—PubMed began to selectively index Covid preprints in June 2020 (studies with NIH support) and Elsevier made preprints from medRxiv and bioRxiv available in Embase from January 2022. Additionally, the announcement of major trial results via media releases often provided important context for guideline panel deliberations, even if the absence of published data meant the results could not directly inform recommendations. In response, we added searches of preprint servers to our surveillance, tracked key trial websites, and relied on the evidence team's monitoring of social media to capture breaking news.

The rush to publish, spurred on by the urgency of the pandemic, has been held partly responsible for the high rate of studies that were subject to expressions of concern or retracted [17]. A high‐profile example was the registry analysis of hydroxychloroquine for treating COVID‐19 published in *The Lancet* in May 2020 and retracted within a matter of weeks when the integrity of the data was questioned [18].

This and other early examples emphasized the importance of checking for retractions as part of routine surveillance, as well as being aware of commentary highlighting concerns with studies in general—this was particularly the case for ivermectin [19] and hydroxychloroquine, which resulted in several studies being removed from the guidelines as methodological concerns came to light. A PubMed auto alert notified us of errata, expressions of concern and retracted publications, and we subscribed to the RSS feed from Retraction Watch (https://retractionwatch.com/).

## 4 ALL SYSTEMS ARE GO

By May 2020, as the pace of published studies picked up and the guidelines took shape, the initial frenetic period gave way to a more steady and systematic approach to evidence surveillance. Since the guidelines focused on the care of people with COVID‐19, and thus excluded topics relating to testing, vaccines, or public health measures to prevent the spread of the disease, randomized trials became the principal source of evidence for informing treatment recommendations. This greatly simplified the surveillance approach, since monitoring sources for randomized trials was relatively straightforward compared, for example, with searching for studies relevant to diagnosis or prognosis.

For those sections of the guideline that included recommendations unlikely to be informed solely by randomized trials (e.g., care of pregnant and breastfeeding women, or different aspects of mechanical ventilation) other evidence was considered. However, instead of conducting our own searches and syntheses of these studies, our recommendations were informed by regularly updated living systematic reviews (LSRs) that other reputable groups internationally were producing. Examples included the PregCOV‐19 LSR Consortium at the University of Birmingham, UK [20] and a WHO‐sponsored LSR of ventilation techniques [21]. An early surveillance activity thus involved compiling a list of living reviews to monitor for updates.

At around this time, as the volume of research escalated, using the CDC database of daily downloadable citations combined with screening in EndNote to monitor new evidence became increasingly impractical. In its place we used the WHO COVID‐19 Database (which by this time was also receiving the daily feed of citations from CDC) for records of possible trials added each day, using the sort by entry date function. This continued until early 2021 when we dropped the daily search because the database was no longer uniquely identifying studies that we were not already aware of.

Throughout the pandemic the mainstay of search surveillance was PubMed, medRxiv preprint server, and the COVID‐19 collections of key journals. Daily and weekly auto alerts in PubMed were set up to identify randomized trials, systematic reviews, and research relevant to specific topics or populations covered by the guidelines. The COVID‐19 SARS‐CoV‐2 preprints from medRxiv were also scanned daily. A search of another preprint server, Research Square, was superseded by daily searches of Europe PMC in June 2021. Table 1 summarizes the sources that were routinely searched.

**Table 1:** Summary of COVID‐19 evidence surveillance.

Source/site	Action and frequency
WHO COVID‐19 Database	Daily search ((tw:(trial)) OR (tw:(random))) ordered by entry date and manual scan of newly added records. Daily search discontinued in early 2021.
PubMed	Daily auto alerts for randomized trials, systematic reviews, and retractions; weekly auto alerts for respiratory support, pregnancy and newborn care, and pediatric inflammatory multisystem syndrome (PIMS‐TS)
Preprints	Daily scan of articles posted to the COVID‐19 SARS‐CoV‐2 preprints section of medRxiv. Daily scan of COVID‐19 preprints from Research Square until June 2021, superseded by Europe PMC search.
Europe PMC	Daily auto alert for randomized trials, limited to preprints as the source
Collections of COVID‐19 research	Daily scan of articles, news pieces, editorials, commentaries, and so forth, added to COVID‐19 collections from JAMA, The Lancet, BMJ, NEJM, Annals of Internal Medicine and Nature
Collections of COVID‐19 syntheses	Daily scan of VA COVID‐19 Evidence Reviews, Oxford COVID‐19 Evidence Service, COVID‐END, COVID‐19 Rapid Evidence Reviews (NCCMT, Canada), NICE. Daily search discontinued in early 2021; sources searched in response to new guideline topics or questions.
RSS Feed	Push notifications received from Retraction Watch and platform trial sites (RECOVERY, PRINCIPLE, PANORAMIC)
Cochrane	Daily check of Cochrane Slack channel for new or updated Cochrane reviews
COVID‐NMA	Cross‐check of randomized trials newly identified by covid-nma.com (initially daily then weekly followed by fortnightly as the update schedule evolved)
NSW Health (Australia)	Daily scan of evidence digest from COVID‐19 Critical Intelligence Unit for items relevant to the Australian context
Social media	Evidence team continually monitor Twitter for breaking news of new studies

During 2020 the PubMed and medRxiv searches were supplemented by several other sources that were regularly updated with evidence or information unlikely to be retrieved from traditional databases or preprint servers; these included syntheses and rapid guidance from groups such as National Institute for Health and Care Excellence (NICE) (UK), Oxford COVID‐19 Evidence Service, COVID‐19 Rapid Evidence Reviews (Canada), and the collection of evidence reviews maintained by the VA Evidence Synthesis Program.

Identifying syntheses and guidance from these groups served several purposes, including comparing consistency of recommendations, checking that we had captured all relevant studies, and informing specific recommendations for populations not directly covered by our searches (e.g., care of people with co‐existing morbidities such as asthma).

## 5 TIMING IS EVERYTHING

**Figure 1:**
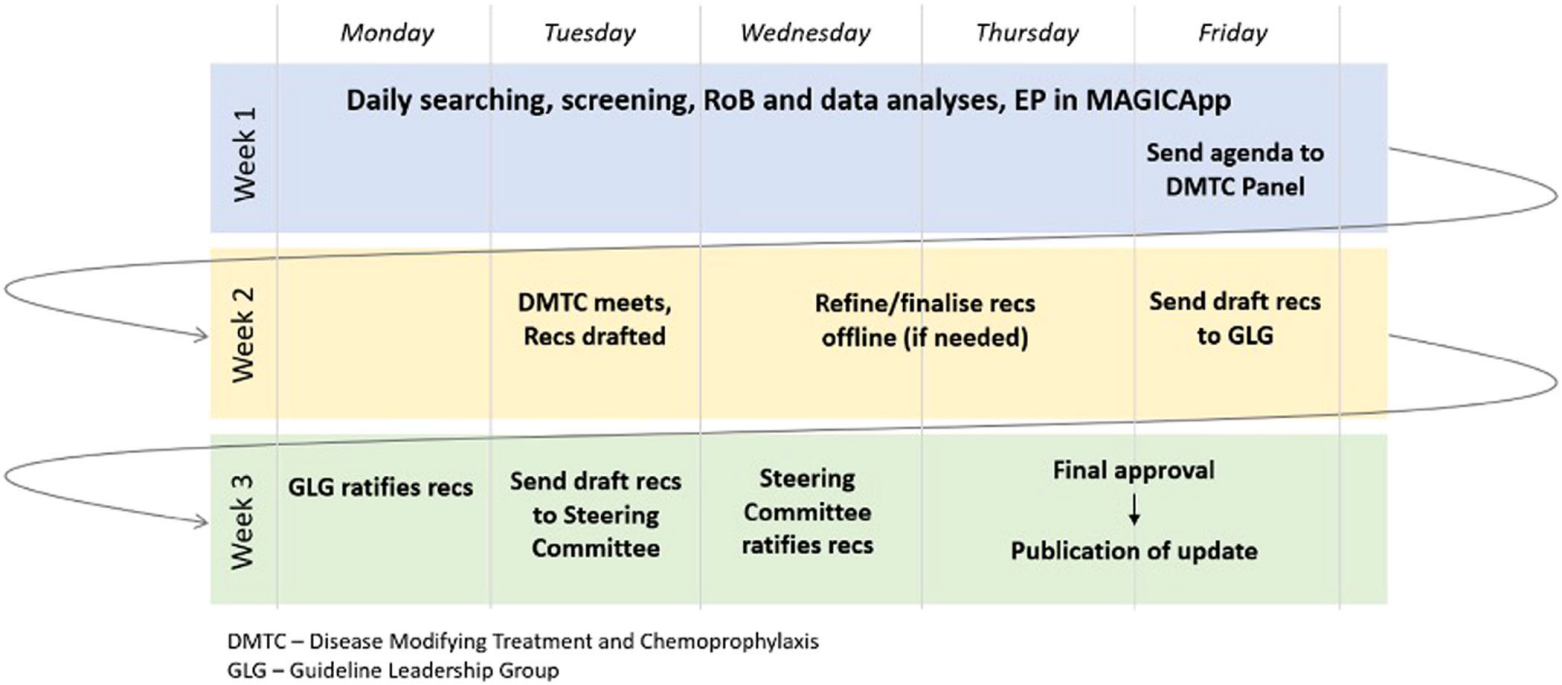
Taskforce guidelines: development and publication cycle.

Although it might have been feasible to rely on a single source of COVID‐19 research, such as the WHO database, to capture all new studies, the Taskforce's rapid cycle of recommendation development and guideline publication relied on identifying studies as soon as they were published. The knock‐on effect of even a day's delay could have resulted in new or updated recommendations missing that week's development and publication cycle (Figure 1). (The guideline development process is described fully in an earlier paper [3].)

**Figure 2:**
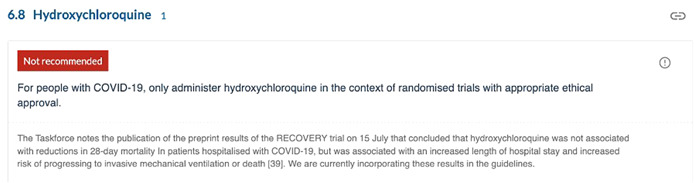
Screenshot of Taskforce guidelines (version 13, July 16, 2020) showing inclusion of RECOVERY preprint for hydroxychloroquine, published July 15, 2020.

Daily checking of medRxiv and the website collections of COVID‐19 articles from publishers such as *BMJ, JAMA*, *The Lancet* and *New England Journal of Medicine* meant that within hours of publication, key studies were in the hands of the evidence team. Articles in the *NEJM* for example were made available online at 7am or 9am Melbourne time, depending on the time of year. Similarly *The Lancet's* standard embargo time of 11.30 p.m. UK time coincided with the routine morning surveillance. On several occasions, the weekly publishing schedule of the guidelines coincided with the publication of critical studies (e.g., RECOVERY trial preprints) and we were able to draw attention to these results in the guidelines in near real‐time, ahead of their formal integration in new or updated recommendations (Figure 2).

As the pandemic continued, it became apparent that studies with the most impact on treatment recommendations were typically first published either as preprints or in the major general medical journals, or their sister journals. As reported in an earlier paper [2], around half the studies (61/129) included in the first 12 months of the COVID‐19 guidelines were published as preprints (mostly in medRxiv), and 50 were published (either initially or ultimately) in *NEJM* (23), *JAMA* (10) *The Lancet* (6), *Lancet Respiratory Medicine* (5), *BMJ* (3), *JAMA Internal Medicine* (2), and *Annals of Internal Medicine* (1).

Several of the critical studies of COVID‐19 treatments came from the RECOVERY platform trial (https://www.recoverytrial.net/), and we set up an RSS feed that generated push notifications for announcements from this and the subsequent PRINCIPLE and PANORAMIC platform trials.

While monitoring medRxiv and the above‐mentioned journal collections was the most efficient and effective means of surveillance of the most influential studies, the PubMed and Europe PMC alerts captured almost all the remaining studies. As a final safeguard, we cross‐checked our list of known studies against the COVID‐NMA site (https://covid-nma.com/) for randomized trials of drug treatments identified from their more exhaustive searches. The site was initially updated daily before moving to weekly and then fortnightly updates in 2021 (and ceasing updates at the end of 2022). The occasional study we uniquely identified from COVID‐NMA tended to be from more obscure (for us) sources (e.g., Russian‐language journals), preprints from SSRN (before Europe PMC was part of daily surveillance), or the results of studies (mostly with small sample sizes) posted directly to ClinicalTrials.gov in the absence of a published report.

## 6 DOCUMENTING CHANGES

We recognized the importance of documenting and making available our search methods but wanted to avoid constantly republishing every time we tweaked a search strategy or sources changed their updating frequency. The evolution of the search methods was documented in a pdf available from the methods and processes section of the guideline and was updated every 6 months or so (Supplementary File 1). It listed the “what, when and how” of the sources searched, including the line‐by‐line search strategies for the different auto alerts. A summary of the main changes to each version of the methods appeared at the beginning of the document.

Search strategies were frequently refined in response to changes in guideline scope and the way terms were used and indexed (Box 1). Surveillance was also adapted in response to emerging concerns that affected existing recommendations. For example, the impact of the Omicron variant and sub‐variants on the effectiveness of previously established treatments such as monoclonal antibodies prompted supplementary searches of in vitro evidence.

Box 1 **PubMed search string for COVID‐19 studies**

Some of the earliest novel coronavirus search strategies included a wide array of terms to capture research published before COVID‐19 became the official name of the disease (e.g., 2019nCoV, “wuhan AND coronavirus”) [22]. Our PubMed search string was regularly updated in response to changes in terminology, indexing practice, and usage. For example, several variations of terms for the novel coronavirus were added as supplementary concept records in PubMed (using the [NM] field label) in February 2020 before the MeSH term COVID‐19 was added to the MeSH thesaurus.

We continually refined the core search string for COVID‐19 to maintain precision, for example, by removing redundant terms (e.g., MeSH term Coronavirus Infections and coronavirinae*[tiab]) and changing free‐text terms from appearing in all fields to title only.

In the most recent revision (below), we dropped the two MeSH terms (COVID‐19 and SARS‐CoV‐2) because they retrieved significant numbers of records that were indirectly related to COVID‐19 (e.g., how the conduct of a clinical trial or provision of medical care was affected by the pandemic). The two MeSH terms were unlikely to retrieve reports of trials not also retrieved by the free‐text terms. Similarly, we excluded records with “during COVID‐19” or “during coronavirus” in the title by making use of PubMed's proximity searching.

*2023 version*: ((coronavirus*[ti] OR covid*[ti] OR sars*[ti]) AND (Clinical Trial[pt] OR trial[ti] OR randomi*[tw] OR randomly[tw] OR placebo[tw])) NOT (Systematic[sb] OR meta‐analysis[ti] OR protocol[ti] OR vaccine*[ti] OR vaccination*[ti] OR “during covid‐19”[ti:~3] OR “during coronavirus”[ti:~3])

This search strategy was designed to be appropriate for the Australian COVID‐19 guidelines and is not intended to be used as a generic search string to identify COVID‐19 studies.

## 7 A DAY IN THE LIFE

**Figure 3:**
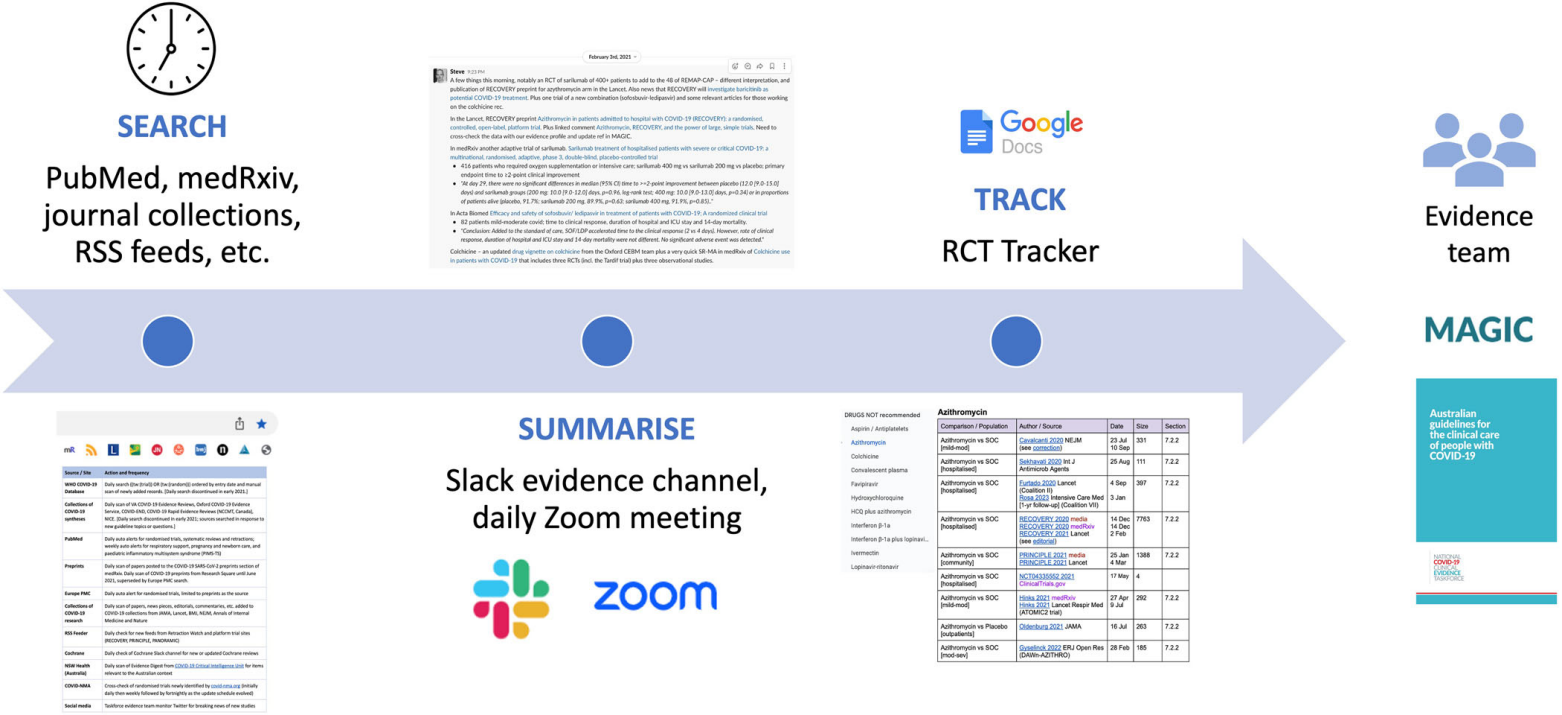
Schema of the surveillance and workflow integration.

What does daily evidence surveillance for a living guideline in a public health emergency look like, and how does it link with the rest of the guideline operation? Most of the evidence team working on the COVID‐19 guidelines were based in Melbourne. At the end of October 2021 when the Victorian Government finally lifted its stay‐at‐home orders, Melbourne had been under six lockdowns since March 2020, totalling 262 days (almost 9 months). There was ample opportunity to hone processes. We relied on tools like Slack and Zoom to facilitate communication and manage day‐to‐day operations (Figure 3).

Daily search surveillance was the responsibility of the information specialist (S. M.) and followed a familiar routine. Records received overnight from the PubMed and Europe PMC alerts were scanned for randomized trials and key systematic reviews. This was followed by checking medRxiv preprints; the COVID‐19 article collections of key journals; RSS feeds; NSW COVID‐19 Critical Intelligence Unit daily digest for information relevant to the Australian context; Cochrane Slack channel for details of newly published COVID‐19 reviews; and the COVID‐NMA site.

**Figure 4:**
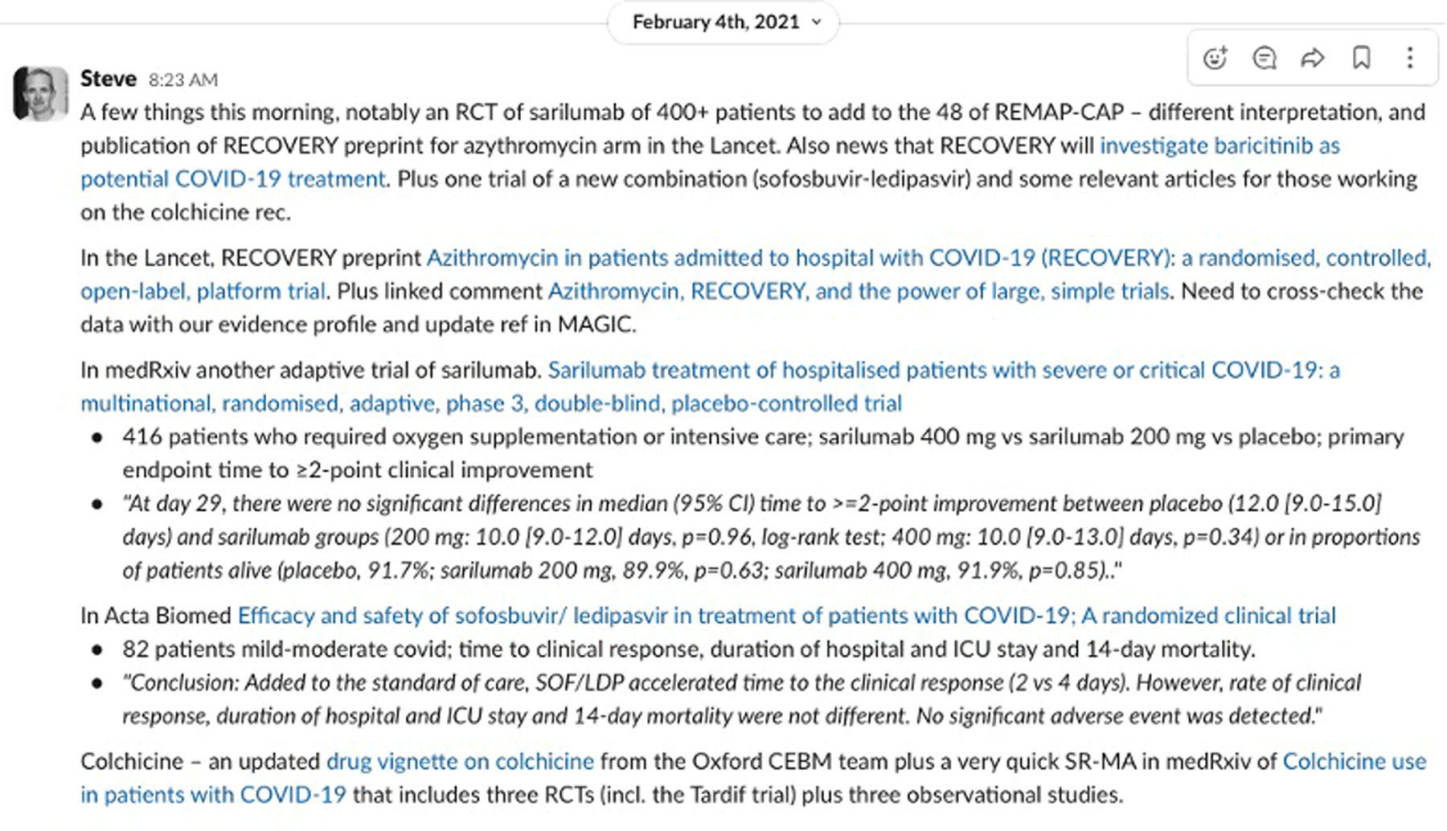
Screenshot of evidence team Slack channel morning digest.

Details of new studies and other information or news of interest were summarized and posted to the evidence team channel in Slack. Description of new studies included basic information (sample size, population, primary outcomes) with a brief extract from the results or conclusions (Figure 4). Items included hyperlinks to the full text or abstract in PubMed. Other information shared by members in the evidence channel included news articles, breaking news, as well as reaction and comment on the information posted.

At 9.30 a.m. weekdays the evidence team met by Zoom for its daily “stand‐up” where that morning's evidence was discussed, tasks allocated and prioritized, and progress reviewed with respect to preparing information and agendas for the various guideline panel meetings.

## 8 WORKFLOW INTEGRATION AND WORKLOAD

Setting up a system that tracked published studies was central to the efficient workflow of the evidence team. We considered tools like Mendeley and Covidence, and later REDCap, for sharing this information but settled on Google Docs. As trials were identified from the daily surveillance (excluding trials of complementary therapies and vaccines, which were out of scope), they were added to the RCT Tracker document. Although this was a relatively simple, low‐tech approach, it was effective in providing the evidence team with an overview of eligible studies, especially at the height of the pandemic when several recommendations were being developed or updated simultaneously.

**Figure 5:**
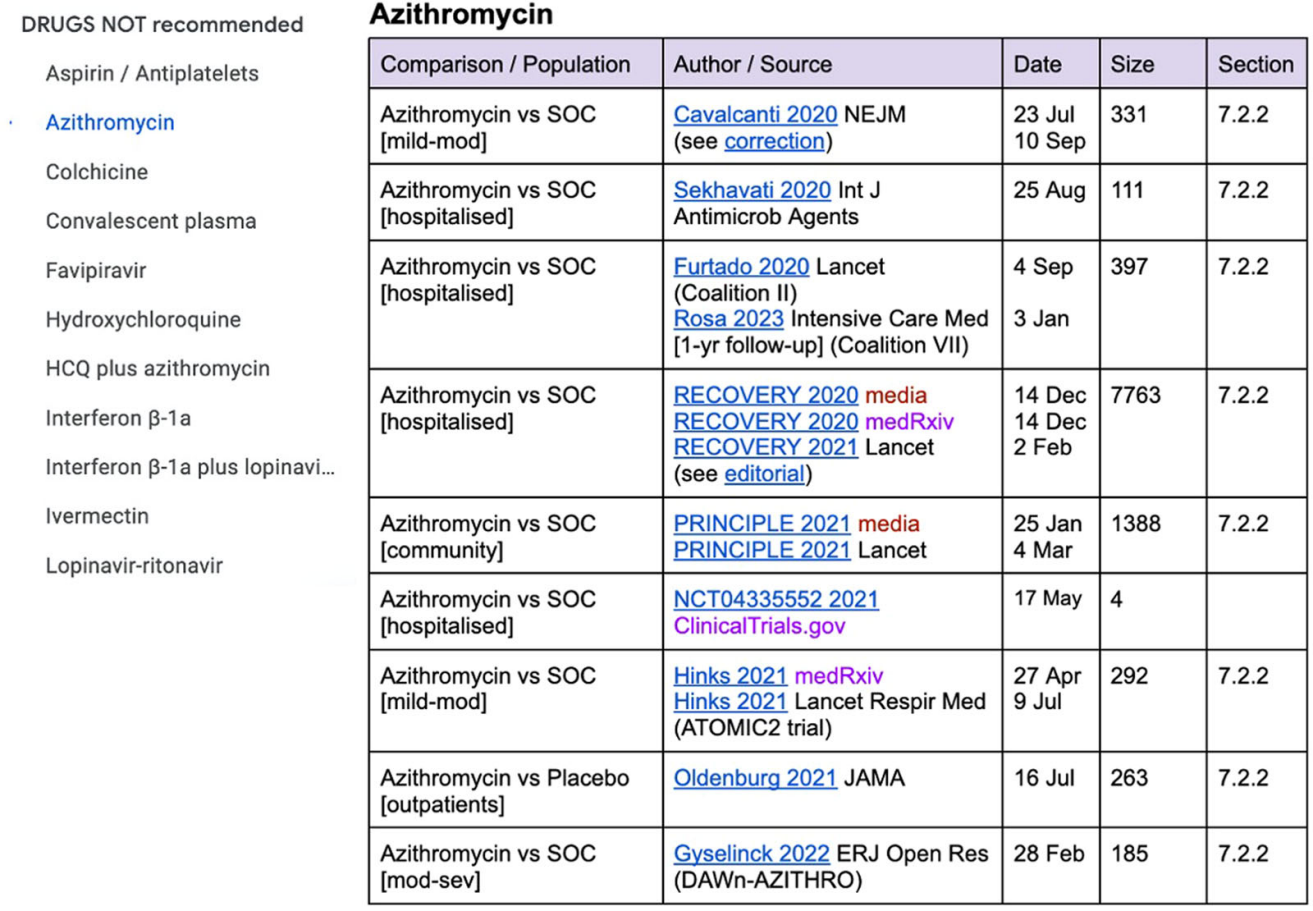
Screenshot of Google Doc used to track trial publications.

The Google Doc matched the structure of the guideline (and was easy to reorganize as sections were added or changed). Details for each study included the comparison (e.g., drug vs. placebo) plus population (e.g., mild‐moderate, hospitalized), the author or trial name, source and date of publication, sample size, and hyperlink to the full text. Different types of study outputs (media releases, preprints, publications, corrections, etc.) and any accompanying editorials or commentaries were linked and presented together in the document. When a study was included in the evidence profile for the guideline recommendation, this was noted in the final column in the table (Figure 5).

One additional task was to add (and where necessary update) references in MAGICapp to studies included in the guidelines. For example, whenever a preprint was published in a journal, the reference would be updated once the evidence team had checked the data and made any revisions to what was already published in the guideline.

The information specialist (S. M.) was responsible for the evidence surveillance, conducting all searches and most of the screening for studies of relevance to the guideline. The exception was the weekly PubMed alerts for the respiratory support, pregnancy and perinatal care, and child and adolescent care sections of the guidelines which were screened in Covidence by the evidence team member responsible for that section.

The workload in managing the surveillance varied according to the stage of the pandemic—during 2020 and 2021 this amounted to about 15 h a week but dropped to 3 h a week in 2023. This included time spent searching and screening, as well as preparing the daily summary in Slack, updating the RCT Tracker, and attending the daily (latterly twice‐weekly) evidence team meetings.

Over the 3 years of daily surveillance, records retrieved by the PubMed search for randomized trials varied from 25 to 30 a day at the peak in 2021 to around five or six records a day in 2023. The systematic reviews alert retrieved around double this number. Since March 2020 we estimate that about 20,000 PubMed records (alerts for trials and reviews) and a similar number of medRxiv records have been scanned.

Australian Government funding for the COVID‐19 guidelines ceased at the end of 2022 and the guideline operations were scaled back accordingly—the evidence team was reduced by two‐thirds, and the guideline panels only convened in response to new studies or information that impacted existing recommendations. We continued with daily surveillance until the end of May 2023 when we switched to weekly searches and limited surveillance to PubMed and Europe PMC alerts only. Search surveillance ceased altogether at the end of September 2023.

## 9 DISCUSSION

The rapidity, volume and breadth of evidence generated during the pandemic created a novel and challenging environment in which to develop clinical guidelines. This article describes how an evidence surveillance service was established to support a living guideline for the care of patients with COVID‐19 that was updated over 70 times, and at the peak of the pandemic was updated weekly. Here we reflect on the feasibility and implications of our experience.

First, feasibility was aided by the prescribed scope of the guidelines. Although broad in coverage—the guidelines contained over 200 recommendations—the focus on treatments meant we could primarily concentrate on identifying reports of randomized trials. This simplified the overall search approach, making the manual screening of records an achievable and straightforward task for a solo information specialist.

In contrast, initiatives with broader scope, for example registers of COVID‐19 research, faced challenges that were addressed through automation, specifically machine learning classifiers [23]. Minimizing screening through automation was also implemented when researchers faced more heterogeneous studies and data, as was the case with the suite of diagnostic test reviews published by Cochrane, or where evidence was monitored across a broad portfolio, as was the case with the living guidelines maintained by NICE [24].

Second, our pragmatic approach combined conventional methods of information retrieval (database searches) with supplementary methods (daily scanning of journal collections, preprint servers, RSS feeds) that addressed the need for urgency and timeliness in identifying evidence likely to have the most clinically important impact on recommendations. Efficiencies were achieved by drawing on secondary sources, such as the COVID‐NMA initiative that were derived from more extensive searches, giving us confidence that we were unlikely to miss relevant studies.

Third, we recognized that it was not feasible or desirable to develop every recommendation in isolation from other groups preparing evidence syntheses. For the less time‐critical sections of the guidelines that drew on different types of evidence (e.g., timing of surgery after a Covid infection, care of patients with comorbidities), we used living syntheses from other groups to inform our recommendations. We also established a collaboration with the evidence team at NICE, meeting monthly to share information and minimize duplication; this was especially useful for long COVID, as we received monthly digests based on NICE's extensive literature searching [25].

Fourth, the speed with which recommendations were developed relied on a well‐functioning integration of search surveillance with the workflow of the evidence team. Clear team roles and responsibilities, the use of messaging apps and communications platforms, such as Slack and Zoom, and tracking studies using Google Docs, proved highly effective in establishing an efficient team environment.

Finally, the advantages of having a single, experienced, committed information specialist designing, running, communicating and coordinating this effort for the entire project period should not be underestimated [26]. Involvement in the process from day one enabled a level of engagement and immersion that is not typical of standard guidelines. For example, being very familiar with the evidence as it accumulated during the pandemic meant the importance and potential impact of new studies could be quickly relayed to the evidence team. Similarly, being aware of the vagaries of Covid sources meant adaptations to surveillance could be made to ensure it remained optimal.

It is reassuring that our approach encapsulated several of the principles published in a recent white paper from the Best Practices for Searching During Public Health Emergencies Working Group [27]. Principles such as timeliness (considering urgency, trade‐offs and efficiencies), balance (combination of new and traditional tools) and responsiveness (maintaining situational awareness and flexibility) underpinned our search surveillance methods.

The exceptional nature of COVID‐19 presented a unique opportunity to test and accelerate the adoption of living evidence surveillance methods [28]. Before Covid our experience with the Australian Stroke Foundation living guidelines [29, 30] was that monthly surveillance was more than adequate—it was inconceivable that daily surveillance would ever need to be an essential component of a living guideline. And outside of a global public health emergency, it is hard to imagine how new evidence could transform practice within days of being made available. We applied a similar living guidelines approach when mpox (monkeypox virus) was declared a public health emergency in mid‐2022. We set up daily auto alerts in PubMed and Europe PMC, and monitored the special collections maintained by the *BMJ* and *The Lancet*. Although this was simple enough to do, mpox never matched the seriousness of COVID‐19 and after four versions the living guidelines were retired in May 2023 [31].

Although the urgency of COVID‐19 was unique, several principles that guided our surveillance are applicable to living guidelines more generally [32]. Understanding what is appropriate in terms of timeliness (frequency), coverage (range and type of sources) and approach (manual vs. automation), and what is feasible given resource constraints (time and personnel) are key considerations. Once living guidelines are established, weighing up the potential benefits and trade‐offs of new sources and tools is an iterative process that ensures the surveillance approach remains fit for purpose.

There is a perception with searching that being pragmatic may not be compatible with being systematic—that there's the potential of missing evidence by taking shortcuts. The example of the COVID‐19 guidelines demonstrates that a tailored, continually adapted approach to evidence surveillance can be as effective in terms of identifying relevant evidence, while being more efficient and consuming considerably fewer resources.

## 10 CONCLUSION

Living evidence became a hallmark of the COVID‐19 pandemic. Australia's living COVID‐19 guidelines demonstrated that daily evidence surveillance for over 3 years was not only feasible but also essential for their success. The continual evolution of the surveillance approach for COVID‐19 exemplified principles applicable to living guidelines more generally, namely the need to adapt to changes in context (scope, timeliness, sources) and resources, and the importance of embedding surveillance in the overall evidence workflow.

## AUTHOR CONTRIBUTIONS

**Steve McDonald**: Conceptualization; methodology; investigation; formal analysis; writing—original draft; writing—review and editing. **Tari Turner**: Conceptualization; supervision; writing—review and editing.

## CONFLICT OF INTEREST STATEMENT

The authors declare no conflicts of interest.

## PEER REVIEW

The peer review history for this article is available at https://www.webofscience.com/api/gateway/wos/peer-review/10.1002/cesm.12045.

## Supporting information

Supporting information.

## Data Availability

Data sharing is not applicable to this article as no new data were created or analyzed in this study.
