# Supplementary material for: Designing, refining and reflecting on 3 years of daily evidence surveillance for Australia's living national COVID‐19 guidelines
Source: Cochrane Evid Synth Methods. 2024 Feb 26;2(3):e12045. doi: 10.1002/cesm.12045 (PMC11795968; doi:10.1002/cesm.12045)
Supplement: Supplementary file 1 — Supporting information. [file CESM-2-e12045-s001.pdf]

Search Methods

Document compiled by	Steve McDonald, Senior Information Specialist
Version and Date	Version 4, 1 October 2022
Main changes from version 1 (Nov 2020)	1. Following sites 1) COVID-19 Evidence Reviews, 2) Oxford CEBM and 3) Canadian Agency for Drugs and Technologies in Health are no longer checked daily but are searched in response to specific PICOs.<br>2. COVID-NMA Initiative checked weekly rather than daily (site is updated every Friday).<br>3. PubMed alerts: 1) replaced NLM supplementary terms for coronavirus with COVID-19[Mesh Terms]. 2) Removed Coronavirus[Mesh Terms] and Coronavirus Infections[MesH terms] since records retrieved by these terms are retrieved by coronavirus*, plus records relevant to COVID-19 are now retrieved with the new MeSH term. 3) Excluded protocols for reviews and meta-analyses from PubMed alerts, in addition to trials. 4) Respiratory support and Pregnancy & newborn PubMed alerts changed from daily to weekly.<br>4. Added two new LSR (macrolides and cell-based therapies)
Main changes from version 2 (Dec 2020)	1. PubMed alerts 1) added SARS-CoV-2[MeshTerms] to all search strategies. 2) Removed 2020:3000[DP], corono**o**virus* and coronavirinae* from all search strategies. 3) Revised Respiratory support and Pregnancy and newborn care search strings to retrieve systematic reviews and trials only (not observational studies). 4) Added tocilizumab, baricitinib, sarilumab, casirivimab, imdevimab and REGEN-COV to the Pregnancy and newborn care search. 5) Aerosol-generating procedures search no longer active. 6) Patient preferences and values search no longer active. 7) Separated RCT and SR PubMed alerts, and amended search to retrieve covid-related terms in the title only.<br>2. Sources searched 1) added PubMed daily alert to Table 2. 2) removed CDC Register from search for new PICOs in Table 4, and added Europe PMC and NIH COVID-19 Portfolio. 3) CEBM removed from weekly check. 4) Research Square and WHO COVID-19 Database removed from daily check in Table 2. 5) Added Europe PMC to sources search on a weekly basis in Table 2.
Main changes from version 3 (Jul 2021)	1. PubMed 1) ceased separate weekly search for respiratory support interventions at the end of February 2022 since only randomised trials are now considered for inclusion (and these are retrieved in the main COVID-19 daily PubMed alert). 2) Daily alert added for retractions,

	errata, etc. related to COVID-19 randomised trials (from November 2021). 3) Daily alert added for *in vitro* studies relating to neutralising activities on selected SARS-CoV-2 omicron subvariants (from March 2022). 4) added meta-analysis[Title] to the list of exclusion terms for the RCTs search (see section 3.1). 5) Added search for risk factors for deterioration (see section 2.5).<br>2. Europe PMC 1) daily automated alerts replaced weekly alerts from 10 November 2021. 2) Daily alert added for *in vitro* studies relating to neutralising activities on selected SARS-CoV-2 omicron subvariants (from March 2022).<br>3. COVID NMA Initiative 1) fortnightly search replaced weekly search since the site is now updated every two weeks (from March 2022) with trials evaluating antivirals and immunomodulators only.<br>4. RSS feeds set up for Retraction Watch and the RECOVERY, PRINCIPLE and PANORAMIC platform trials.<br>5. Added description of search for 'Care after COVID-19' (see 2.5).<br>6. Amended section on searching for living systematic reviews (see 2.2).

1. Introduction

This document outlines our overall approach to evidence surveillance and provides details of the routine sources we consult and the searches we run on a daily, weekly or fortnightly basis to identify evidence relevant to the scope of the guideline.

This version of the document reflects the transition of the guideline to sustain mode from July 2021 and includes further changes made to September 2022.

The aim of the guideline is to provide recommendations on the management and care of people with suspected or confirmed COVID-19. With the exception of chemoprophylaxis for the prevention of infection in people exposed to COVID-19 and the prevention of infection to health professionals using respiratory support interventions, the guideline does not include other interventions (e.g., vaccines) used in the prevention of COVID-19 infection or transmission.

The main sections of the guideline cover drug treatments, chemoprophylaxis, respiratory support, venous thromboembolism prophylaxis, therapies for existing indications, care after COVID-19, timing of surgery following COVID-19 infection, pregnancy and perinatal care, and child and adolescent care.

As the guideline has evolved over the course of the pandemic, the type and sources of evidence used to inform recommendations have become more focused. For drug treatments and chemoprophylaxis, our primary interest is in randomised trials. For the remaining sections, in the absence of randomised trials, we consider non-randomised or observational studies, or high-quality systematic reviews or other evidence synthesis, especially living reviews.

In accordance with our policy, the guideline includes evidence from both published studies and studies that are only available as preprints.

Table 1 summarises the sources we search (and frequency) relevant to the various sections of the guideline, and how the retrieved citations are screened for inclusion. Manually scanned citations are looked at by one person (SM). All new studies, including publications of studies previously published as preprints, and any updates to living systematic reviews and other living syntheses, are notified to the evidence review team for consideration. Citations that are imported into Covidence are screened by members of the evidence review team.

Table 1: Search methods summary

Topic and approach	Source	Frequency
COVID-19 randomised trials[1]<br>In-scope of guideline recommendations	PubMed alert<br>medRxiv<br>Europe PMC (preprints)	Daily<br>Manually scanned
	COVID-NMA Initiative	Fortnightly<br>Manually scanned
COVID-19 systematic reviews<br>In-scope of guideline recommendations	PubMed alert<br>Cochrane (via Slack channel)	Daily<br>Manually scanned
Pregnancy and newborn care<br>C19 + SRs, RCTs	PubMed alert	Weekly → Covidence
PIMS-TS<br>C19 + SRs, RCTs, NSRIs, observational studies	PubMed alert	Weekly → Covidence
Omicron *in vitro* studies	PubMed alert<br>Europe PMC (preprints)	Daily<br>Manually scanned

[1] further details in Table 2

2. Surveillance activities

2.1 Sites checked daily or weekly

The first component of our evidence surveillance activities comprises the daily (Monday through Friday) or weekly checking of several resources and sites for relevant primary studies and reviews (Table 2). The aim is to identify new reports of randomised trials as soon as they are published to allow the evidence review team maximum time to prepare new or updated evidence profiles for consideration by the guideline panels. To safeguard against missing potential studies, we cross-check with the COVID-NMA Initiative site, which undertakes extensive searches to identify all COVID-19-related randomised trials, and provides fortnightly updates of the trials identified. Since March 2022 a daily search has been added to identify *in vitro* studies relating to neutralising activities on selected SARS-CoV-2 omicron subvariants.

Table 2: Sites checked daily, weekly or fortnightly

Coverage	Sources
COVID-19 primary studies	**PubMed** (pubmed.gov)<br>➔ Daily alerts for randomised trials, systematic reviews and retractions<br>➔ Daily alert for *in vitro* studies relating to neutralising activities on selected SARS-CoV-2 omicron subvariants (from March 2022)<br>➔ Weekly alerts for pregnancy and newborn care, and PIMS-TS.
	**medRxiv preprint server** (medrxiv.org)<br>➔ manual scan of all papers posted in the previous 24 hours.
	**Europe PMC** (europepmc.org)<br>Preprints only. Includes preprints from medRxiv, Research Square, preprints.org and SSRN, among others.<br>➔ Daily alerts for randomised trials.<br>➔ Daily alerts for *in vitro* studies relating to neutralising activities on selected SARS-CoV-2 omicron subvariants (from March 2022)
	**COVID-NMA initiative** (covid-nma.org)<br>Identifies randomised trials from daily screening of searches of the L.OVE platform and the Cochrane COVID-19 Study register platform, among others. Provides study characteristics, risk of bias assessments and forest plots. From March 2022 the site only lists trials evaluating antivirals and immunomodulators.<br>➔ Check the site's fortnightly update every other Monday
	**RSS Feeds**<br>Notification of items that appear in Retraction Watch plus news alerts from the RECOVERY, PRINCIPLE and PANORAMIC platform trials.<br>➔ Checked daily
	**Collections of COVID-19 articles from leading journals**<br>➔ Lancet, JAMA, BMJ, NEJM sites checked daily
Other sources	**NSW Health** COVID-19 Critical Intelligence Unit Daily Evidence Digest checked weekly for relevant studies or commentaries.
Social media	Members of the evidence review team monitor Twitter for breaking news of new studies.

2.2 Living systematic reviews (LSRs)

We had previously identified relevant living systematic reviews and developed a specific PubMed search to monitor and retrieve updates. These LSRs were rarely a source of trials that we are unaware of, but were occasionally useful for comparing approaches in how evidence is assessed and interpreted.

At this stage in the pandemic (September 2022) only a few of these LSRs are being regularly updated. We no longer have a specific LSR search but continue to identify living reviews and any updates through our daily PubMed search. The suite of COVID-19 reviews maintained by Cochrane and the two BMJ-published reviews (*Drug treatments for covid-19: living systematic review and network meta-analysis* and *A living guideline on drugs for covid-19*) are the key living reviews we continue to monitor.

2.3 Preprints

In addition to our daily manual search of medRxiv, a daily automated alert identifies preprints from Europe PMC, which collates preprints from several preprint servers.

**Europe PMC**<br><br>RCTs	((TITLE:"covid") OR (TITLE:"covid-19") OR (TITLE:"coronavirus") OR (TITLE:"sars-cov-2") OR (TITLE:"sars")) AND (((TITLE:"trial") OR (ABSTRACT:"trial") OR (TITLE:"randomised") OR (ABSTRACT:"randomised") OR (TITLE:"randomized") OR (ABSTRACT:"randomized") OR (ABSTRACT:”randomly”)) NOT ((TITLE:"vaccine") OR (TITLE:"vaccines") OR (TITLE:"protocol") OR (TITLE:"meta-analysis"))) AND (SRC:PPR)
in vitro studies	((TITLE:"omicron" OR (TITLE:"BA4") OR (TITLE:"BA5") OR (TITLE:"BA.4") OR (TITLE:"BA.5") OR (TITLE:"BA4/5") OR (TITLE:"BA.4/5") OR (TITLE:"BA2.75") OR (TITLE:"BA.2.75")) AND ((TITLE:"mabs") OR (TITLE:"antibody") OR (TITLE:"antibodies") OR (TITLE:"neutralising") OR (TITLE:"neutralizing") OR (TITLE:"neutralisation") OR (TITLE:"neutralization") OR (TITLE:"vitro") OR (TITLE:"in-vitro") OR (TITLE:"sotrovimab") OR (TITLE:"casirivimab") OR (TITLE:"imdevimab") OR (TITLE:"tixagevimab") OR (TITLE:"cilgavimab")) AND (SRC:PPR))

2.4 COVID-19 core search strings for PubMed

Our PubMed searches are based on core search strings for COVID-19 and study design terms, which are then combined with topic-specific terms (see Section 3). These search strings were last amended on 1 July 2021. Search strings for retractions and *in vitro* studies were added in November 2021 and March 2022, respectively.

**COVID-19 terms**<br><br>Free-text terms all fields	(COVID-19[Mesh Terms] OR SARS-CoV-2[Mesh Terms] OR coronavirus* OR "corona virus" OR COVID OR COVID-19 OR COVID19 OR sars* OR "severe acute respiratory syndrome*")

**Study design terms**<br><br>MeSH terms exploded; SRs, RCTs, observational studies, excluding trial and review protocols	Systematic[SB] OR meta-analysis[Publication Type] OR Cochrane Database Syst Rev[SO] OR review[Title] OR "systematic review"[Text Word] OR "rapid review"[Text Word] OR "scoping review"[Text Word] OR "living review"[Text Word] OR "meta-analysis"[Text Word]<br><br>Clinical Trial[Publication Type] OR trial[Title] OR randomized[Text Word] OR randomised[Text Word] OR randomly[Text Word] OR placebo[Text Word]<br><br>Epidemiologic Studies[Mesh] OR Observational Study[Publication Type] OR Evaluation Study[Publication Type] OR Comparative Study[Publication Type] OR study[Title] OR analysis[Title] OR cohort[Text Word] OR "case series"[Text Word] OR "case reports"[Text Word] OR cross-section*[Text Word] OR "retrospective analysis"[Text Word] OR non-randomised[Text Word] OR non-randomized[Text Word] OR case-control*[Text Word] OR "controlled before*"[Text Word] OR "control group"[Text Word] OR "prospective study"[Text Word] OR "retrospective study"[Text Word] OR "observational study"[Text Word] OR guideline*[Text Word]<br><br>NOT (protocol[Title] AND (randomi*[Title] OR review[Title] OR meta-analysis[Title]))
**Retractions**<br><br>Specific to RCTs	(((COVID-19[MeSH Terms] OR SARS-COV-2[Mesh Terms] OR coronavirus*[Title] OR covid*[Title] OR sars*[Title]) AND (Clinical Trial[Publication Type] OR trial[Title] OR randomi*[Text Word] OR randomly[Text Word] OR placebo[Text Word])) NOT (Systematic[SB] OR protocol[Title] OR vaccine[Title] OR vaccines[Title])) AND ((Expression of Concern[PT] OR Corrected and Republished Article[PT] OR Published Erratum[PT] OR Retracted Publication[PT] OR Retraction of Publication[PT]))
***in vitro* studies**<br><br>Specific to omicron subvariants	(omicron[ti] OR BA4[TI] OR BA5[TI] OR BA.4[TI] OR BA.5[TI] OR BA4/5[TI] OR BA.4/5[TI] OR BA2.75[TI] OR BA.2.75[TI]) AND (mabs[ti] OR antibod*[ti] OR neutral*[ti] OR vitro[TI] OR in-vitro[TI] OR sotrovimab[ti] OR casirivimab[ti] OR imdevimab[ti] OR tixagevimab[ti] OR cilgavimab[ti])

2.5 Other searches, sites and sources

For the section of the guideline 'Care after COVID-19' we receive monthly evidence summaries from the National Institute for Health and Care Excellence (NICE) in the UK. These evidence summaries are derived from weekly surveillance searches that NICE conducts for their guideline 'Managing the long term effects of COVID-19 (NG188)'.

For the section of the guideline 'Monitoring and markers of clinical deterioration' we run the following PubMed search (backdated to May 2021). Latest search was run on 26 August 2022.

> (models[ti] OR index[ti] OR tool*[ti] OR predict*[ti] OR score*[ti] OR status[ti] OR risk[ti] OR factors[ti]) AND (morbidity[ti] OR mortality[ti] OR death*[ti] OR fatal*[ti] OR disease[ti] OR severe[ti] OR severity[ti] OR prognos*[ti] OR critical*[ti] OR progression[ti] OR ICU[ti] OR ventilat*[ti] OR "respiratory failure"[ti]) AND (covid*[ti] OR coronavirus[ti]) AND 2021/05/01:3000/12/12[pdat] AND english[Filter]

Whenever new PICOs are considered or developed for the guideline, we run tailored searches of the following sources (Table 3).

Table 3: Sites checked for new PICOs

Type	Sources
Repositories of COVID-19 research, producers of evidence summaries/reviews	WHO COVID-19 Database<br>Cochrane COVID-19 Study Register<br>L·OVE COVID-19 Evidence<br>NIH COVID-19 Portfolio<br>COVID-19 Rapid Evidence Reviews<br>COVID-19 Evidence Reviews<br>PubMed<br>Europe PMC

3. PubMed searches and alerts

PubMed alerts have been set up to cover the various sections of the guideline. These alerts combine the core search strings for COVID-19 and study designs (modified as indicated) with topic-specific terms. In the tables below, the different coloured sections are combined with the Boolean operator AND. Below each table is the search strategy as applied in PubMed.

3.1 All COVID-19 SRs and RCTs

Last amended 1 October 2021

Concept	Search terms
**COVID-19 terms**<br>MeSH terms and title words	COVID-19[Mesh Terms] OR SARS-CoV-2[Mesh Terms] OR coronavirus*[Title] OR covid*[Title] OR sars*[Title])

**Study design terms**<br><br>SRs not protocols or vaccine	(Systematic[SB] OR meta-analysis[Publication Type] OR Cochrane Database Syst Rev[SO] OR review[Title] OR "systematic review"[Text Word] OR "rapid review"[Text Word] OR "scoping review"[Text Word] OR "living review"[Text Word] OR "meta-analysis"[Text Word])) NOT (protocol[Title] OR vaccin*[Title])
RCTs not SRs, protocols or vaccine	(Clinical Trial[Publication Type] OR trial[Title] OR randomi*[Text Word] OR randomly[Text Word] OR placebo[Text Word])) NOT (Systematic[SB] OR meta-analysis[Title] OR protocol[Title] OR vaccine*[Title])

PubMed daily alert SRs (scanned only, not added to Covidence)
((COVID-19[Mesh Terms] OR SARS-CoV-2[Mesh Terms] OR coronavirus*[Title] OR covid*[Title] OR sars*[Title]) AND (Systematic[SB] OR meta-analysis[Publication Type] OR Cochrane Database Syst Rev[SO] OR review[Title] OR "systematic review"[Text Word] OR "rapid review"[Text Word] OR "scoping review"[Text Word] OR "living review"[Text Word] OR "meta-analysis"[Text Word])) NOT (protocol[Title] OR vaccin*[Title])

PubMed daily alert RCTs (scanned only, not added to Covidence)
((COVID-19[MeSH Terms] OR SARS-CoV-2[Mesh Terms] OR coronavirus*[Title] OR covid*[Title] OR sars*[Title]) AND (Clinical Trial[Publication Type] OR trial[Title] OR randomi*[Text Word] OR randomly[Text Word] OR placebo[Text Word])) NOT (Systematic[SB] OR meta-analysis[Title] OR protocol[Title] OR vaccine*[Title])

3.2 Pregnancy and newborn care

Last amended on 1 July 2021

Concept	Search terms
**COVID-19 terms**	(COVID-19[Mesh Terms] OR SARS-CoV-2[Mesh Terms] OR coronavirus* "corona virus" OR COVID OR COVID-19 OR COVID19 OR sars* OR "severe acute respiratory syndrome*")
**Pregnancy MeSH Terms**<br><br>All terms exploded	Pregnancy[Mesh Terms] OR Pregnancy Complications[Mesh Terms] OR Maternal Health[Mesh Terms] OR Perinatal Care[Mesh Terms] OR Prenatal Care[Mesh Terms] OR Postpartum Period[Mesh Terms] OR Infant, Newborn[Mesh Terms] OR Infant, Newborn, Diseases[Mesh Terms] OR Infectious Disease Transmission, Vertical[Mesh Terms] OR Breast Feeding[Mesh Terms]
Pregnancy terms in Title	pregnan*[Title] OR mother*[Title] OR maternal[Title] OR obstetric*[Title] OR birth*[Title] OR perinatal[Title] OR newborn*[Title] OR neonat*[Title] OR infant*[Title]

Breastfeeding and rooming-in	breastfeed*[Text Word] OR "breast feed*"[Text Word] OR breastfed[Text Word] OR "breast fed"[Text Word] OR kangaroo[Text Word] OR skin-to-skin[Text Word] OR ((rooming*[Text Word] OR isolat*[Text Word] OR separat*[Text Word]) AND (newborn*[Text Word] OR baby[Text Word] OR babies[Text Word] OR infant*[Text Word] OR neonate*[Text Word] OR mother*[Text Word] OR maternal*[Text Word]))
Vertical transmission	vertical[Title] OR "vertical transmission"[Text Word] OR mother-to-child[Text Word] OR mother-to-fetal[Text Word] OR maternal-fetal[Text Word] OR maternal-child[Text Word] OR ((mother[TIAB] OR maternal[TIAB] OR woman[TIAB] OR women[TIAB) AND (transmission[TIAB] OR transmit*[TIAB) AND (fetus[TIAB] OR foetus[TIAB] OR fetal[TIAB] OR baby[TIAB] OR newborn[TIAB] OR neonate[TIAB))
Breast milk, breast cleansing, cord clamping	"human milk"[Text Word] OR "breast milk"[Text Word] OR breastmilk[Text Word] OR “breast cleansing”[Text Word] OR “cord clamping”[Text Word] OR “water birth*”[Text Word]
Antenatal steroids, ACE inhibitors	((antenatal*[Text Word] OR prenatal*[Text Word]) AND (steroid*[Text Word] OR corticosteroid*[Text Word])) OR ((Angiotensin-Converting Enzyme Inhibitors[Mesh Terms] OR Antihypertensive Agents[Mesh Terms] OR Angiotensin Receptor Antagonists[Mesh Terms] OR Peptidyl-Dipeptidase A[Mesh Terms] OR angiotensin OR inhibitors OR ACE-I* OR ACEI* OR ARBs OR antihypertens* OR anti-hypertens* OR hyperten*) AND (Pregnancy[Mesh Terms] OR Postpartum Period[MeSH Terms] OR pregnan* OR post-partum OR postpartum))
Proning, ECMO and anticoagulation	(pregnan*[Text Word] AND (prone[Text Word] OR proning[Text Word] OR extracorporeal[Text Word] OR extra-corporeal[Text Word] OR ECMO[Text Word] OR anticoagulant*[Text Word] OR anticoagulation[Text Word]))
**Study design terms**<br><br>SRs and RCTs<br><br>Not protocols	((Systematic[SB] OR meta-analysis[Publication Type] OR Cochrane Database Syst Rev[SO] OR review[Title] OR "systematic review"[Text Word] OR "rapid review"[Text Word] OR "scoping review"[Text Word] OR "living review"[Text Word] OR "meta-analysis"[Text Word] OR Clinical Trial[Publication Type] OR trial[Title] OR randomized[Text Word] OR randomised[Text Word] OR randomly[Text Word] OR placebo[Text Word]) NOT (protocol[Title] AND (randomi*[Title] OR review[Title] OR meta-analysis[Title])))

PubMed weekly alert (added to Covidence for screening)

From 4 June 2021 amended to limit to reviews and trials only
((COVID-19[Mesh Terms] OR SARS-CoV-2[Mesh Terms] OR coronavirus* OR "corona virus" OR COVID OR COVID-19 OR COVID19 OR sars* OR "severe acute respiratory

syndrome*") AND (Pregnancy[Mesh Terms] OR Pregnancy Complications[Mesh Terms] OR Maternal Health[Mesh Terms] OR Perinatal Care[Mesh Terms] OR Prenatal Care[Mesh Terms] OR Postpartum Period[Mesh Terms] OR Infant, Newborn[Mesh Terms] OR Infant, Newborn, Diseases[Mesh Terms] OR Infectious Disease Transmission, Vertical[Mesh Terms] OR Breast Feeding[Mesh Terms] OR pregnan*[Title] OR mother*[Title] OR maternal[Title] OR obstetric*[Title] OR birth*[Title] OR perinatal[Title] OR newborn*[Title] OR neonat*[Title] OR infant*[Title] OR breastfeed*[Text Word] OR "breast feed*"[Text Word] OR breastfed[Text Word] OR "breast fed"[Text Word] OR kangaroo[Text Word] OR ((rooming*[Text Word] OR isolat*[Text Word] OR separat*[Text Word]) AND (newborn*[Text Word] OR baby[Text Word] OR babies[Text Word] OR infant*[Text Word] OR neonate*[Text Word] OR mother*[Text Word] OR maternal*[Text Word])) OR vertical[Title] OR "vertical transmission"[Text Word] OR mother-to-child[Text Word] OR mother-to-fetal[Text Word] OR maternal-fetal[Text Word] OR maternal-child[Text Word] OR ((mother[TIAB] OR maternal[TIAB] OR woman[TIAB] OR women[TIAB) AND (transmission[TIAB] OR transmit*[TIAB) AND (fetus[TIAB] OR foetus[TIAB] OR fetal[TIAB] OR baby[TIAB] OR newborn[TIAB] OR neonate[TIAB)) OR "human milk"[Text Word] OR "breast milk"[Text Word] OR breastmilk[Text Word] OR skin-to-skin[Text Word] OR "breast cleansing"[Text Word] OR "cord clamping"[Text Word] OR "water birth*"[Text Word] OR ((antenatal*[Text Word] OR prenatal*[Text Word]) AND (steroid*[Text Word] OR corticosteroid*[Text Word])) OR ((Angiotensin-Converting Enzyme Inhibitors[Mesh Terms] OR Antihypertensive Agents[Mesh Terms] OR Angiotensin Receptor Antagonists[Mesh Terms] OR Peptidyl-Dipeptidase A[Mesh Terms] OR angiotensin OR inhibitors OR ACE-I* OR ACEI* OR ARBs OR antihypertens* OR anti-hypertens* OR hyperten*) AND (Pregnancy[Mesh Terms] OR Postpartum Period[MeSH Terms] OR pregnan* OR post-partum OR postpartum)) OR (pregnan*[Text Word] AND (prone[Text Word] OR proning[Text Word] OR extracorporeal[Text Word] OR extra-corporeal[Text Word] OR ECMO[Text Word] OR anticoagulant*[Text Word] OR anticoagulation[Text Word]))) AND ((Systematic[SB] OR meta-analysis[Publication Type] OR Cochrane Database Syst Rev[SO] OR review[Title] OR "systematic review"[Text Word] OR "rapid review"[Text Word] OR "scoping review"[Text Word] OR "living review"[Text Word] OR "meta-analysis"[Text Word] OR Clinical Trial[Publication Type] OR trial[Title] OR randomized[Text Word] OR randomised[Text Word] OR randomly[Text Word] OR placebo[Text Word]) NOT (protocol[Title] AND (randomi*[Title] OR review[Title] OR meta-analysis[Title]))))

In July 2021 the search was revised so that records about treatments recommended in the guidelines (i.e. remdesivir, corticosteroids, tocilizumab, sarilumab, baricitinib, etc.) are retrieved irrespective of inclusion of study design terms. This component of the search has been added to the end of the PubMed alert search using OR, and is updated to include new drugs as treatments are recommended. Last amended 30 September 2022

COVID-19 terms AND (Pregnancy MeSH OR pregnancy etc in title) AND (remdesivr OR	(COVID-19[Mesh Terms] OR OR SARS-CoV-2[Mesh Terms] OR coronavirus* OR coronovirus* OR "corona virus" OR coronavirinae* OR COVID OR COVID-19 OR COVID19 OR sars* OR "severe acute respiratory syndrome*") **AND** (Pregnancy[Mesh Terms] OR Pregnancy Complications[Mesh Terms] OR Maternal Health[Mesh Terms] OR Perinatal Care[Mesh Terms] OR Prenatal Care[Mesh Terms] OR

steroids, etc. in title) plus magnesium sulphate	Postpartum Period[Mesh Terms] OR Infant, Newborn[Mesh Terms] OR Infant, Newborn, Diseases[Mesh Terms] OR Infectious Disease Transmission, Vertical[Mesh Terms] OR Breast Feeding[Mesh Terms] OR pregnan*[Title] OR mother*[Title] OR maternal[Title] OR obstetric*[Title] OR birth*[Title] OR perinatal[Title] OR newborn*[Title] OR neonat*[Title] OR infant*[Title]) **AND** (remdesivir[Title] OR veklury[Title] OR dexamethasone[Title] OR hydrocortisone[Title] OR methylprednisolone[Title] OR steroid*[Title] OR corticosteroid*[Title] OR tocilizumab[Title] OR baricitinib[Title] OR sarilumab[Title] OR antagonist*[Title] OR interleukin[Title] OR casirivimab[Title] OR imdevimab[Title] OR REGEN-COV[Title] OR ronapreve[Title] OR sotrovimab[Title] OR xevudy[Title] OR Magnesium Sulfate[Mesh Terms] OR "magnesium sulfate"[Text Word] OR "magnesium sulphate"[Text Word] OR MgSO4[Text Word] OR budesonide[Title] OR immunomodulat*[Title] OR molnupiravir[Title] OR lagevrio[Title] OR nirmatrelvir[Title] OR paxlovid[Title] OR tixagevimab[Title] OR cilgavimab[Title] OR evusheld[Title] OR regdanvimab[Title] OR regkirona[Title])

3.4 PIMS-TS and MIS-C

Last amended 1 July 2021

Concept	Search terms
**COVID-19 terms**	(COVID-19[Mesh Terms] OR SARS-CoV-2[Mesh Terms] OR coronavirus* OR "corona virus" OR COVID OR COVID-19 OR COVID19 OR sars* OR "severe acute respiratory syndrome*")
PIMS-TS MeSH Terms<br><br>All terms exploded	((Systemic Inflammatory Response Syndrome[MH]) AND (Infant[MH] OR Child[MH] OR Adolescent[MH]))
PIMS-TS free-text terms all fields	PIMS* OR MIS-C OR kawasaki* OR ((multi-system OR multisystem OR "toxic shock syndrome" OR "septic shock syndrome") AND (infant* OR child* OR adolescent* OR paediatric* OR pediatric* OR young*))
**Study design terms**<br><br>SRs, RCTs, NSRIs, observational studies<br><br>Not protocols	((Systematic[SB] OR meta-analysis[Publication Type] OR Cochrane Database Syst Rev[SO] OR review[Title] OR "systematic review"[Text Word] OR "rapid review"[Text Word] OR "scoping review"[Text Word] OR "living review"[Text Word] OR "meta-analysis"[Text Word] OR Clinical Trial[Publication Type] OR trial[Title] OR randomized[Text Word] OR randomised[Text Word] OR randomly[Text Word] OR placebo[Text Word] OR Epidemiologic Studies[Mesh] OR Observational Study[Publication Type] OR Evaluation Study[Publication Type] OR Comparative Study[Publication Type] OR study[Title] OR analysis[Title] OR cohort[Text

	Word] OR "case series"[Text Word] OR "case report*"[Text Word] OR cross-section*[Text Word] OR "retrospective analysis"[Text Word] OR non-randomised[Text Word] OR non-randomized[Text Word] OR case-control*[Text Word] OR "controlled before*"[Text Word] OR "control group"[Text Word] OR "prospective study"[Text Word] OR "retrospective study"[Text Word] OR “observational study”[Text Word] OR guideline*[Text Word]) NOT (protocol[Title] AND (randomi*[Title] OR review[Title] OR meta-analysis[Title])))

PubMed weekly alert (added to Covidence for screening)
(COVID-19[Mesh Terms] OR SARS-CoV-2[Mesh Terms] OR coronavirus* OR "corona virus" OR COVID OR COVID-19 OR COVID19 OR sars* OR "severe acute respiratory syndrome*") AND (((Systemic Inflammatory Response Syndrome[MH]) AND (Infant[MH] OR Child[MH] OR Adolescent[MH])) OR PIMS* OR MIS-C OR kawasaki* OR ((multi-system OR multisystem OR "toxic shock syndrome" OR "septic shock syndrome") AND (infant* OR child* OR adolescent* OR paediatric* OR pediatric* OR young*))) AND (Systematic[SB] OR meta-analysis[Publication Type] OR Cochrane Database Syst Rev[SO] OR review[Title] OR "systematic review"[Text Word] OR "rapid review"[Text Word] OR "scoping review"[Text Word] OR "living review"[Text Word] OR "meta-analysis"[Text Word] OR Clinical Trial[Publication Type] OR trial[Title] OR randomized[Text Word] OR randomised[Text Word] OR randomly[Text Word] OR placebo[Text Word] OR Epidemiologic Studies[Mesh] OR Observational Study[Publication Type] OR Evaluation Study[Publication Type] OR Comparative Study[Publication Type] OR study[Title] OR analysis[Title] OR cohort[Text Word] OR "case series"[Text Word] OR "case report*"[Text Word] OR cross-section*[Text Word] OR "retrospective analysis"[Text Word] OR non-randomised[Text Word] OR non-randomized[Text Word] OR case-control*[Text Word] OR "controlled before*"[Text Word] OR "control group"[Text Word] OR "prospective study"[Text Word] OR "retrospective study"[Text Word] OR "observational study"[Text Word] OR guideline*[Text Word]) NOT (protocol[Title] AND (randomi*[Title] OR review[Title] OR meta-analysis[Title])))
